# Tissue-Wide Expression of Genes Related to Vitamin D Metabolism and FGF23 Signaling following Variable Phosphorus Intake in Pigs

**DOI:** 10.3390/metabo12080729

**Published:** 2022-08-06

**Authors:** Maruf Hasan, Michael Oster, Henry Reyer, Siriluck Ponsuksili, Eduard Murani, Petra Wolf, Dagmar-Christiane Fischer, Klaus Wimmers

**Affiliations:** 1 Research Institute for Farm Animal Biology (FBN), Wilhelm-Stahl-Allee 2, 18196 Dummerstorf, Germany; 2 Faculty of Agricultural and Environmental Sciences, University of Rostock, Justus-von-Liebig-Weg 6b, 18059 Rostock, Germany; 3 Department of Pediatrics, Rostock University Hospital, Ernst-Heydemann-Str. 8, 18057 Rostock, Germany

**Keywords:** animal health, gene expression, mineral homeostasis, non-renal calcitriol production, pigs, tissue specificity

## Abstract

Calcium (Ca) and phosphorus (P) homeostasis is maintained by several regulators, including vitamin D and fibroblast growth factor 23 (FGF23), and their tissue-specific activation and signaling cascades. In this study, the tissue-wide expression of key genes linked to vitamin D metabolism (*CYP2R1*, *CYP27A1*, *CYP27B1*, *CYP24A1*, *GC*, *VDR*) and FGF23 signaling (*FGF23*, *FGFR1-4*, *KL*) were investigated in pigs fed conventional (trial 1) and divergent P diets (trial 2). The tissue set comprised kidney, liver, bone, lung, aorta, and gastrointestinal tract sections. Expression patterns revealed that non-renal tissues and cells (NRTC) express genes to form active vitamin D [1,25(OH)_2_D_3_] according to site-specific requirements. A low P diet resulted in higher serum calcitriol and increased *CYP24A1* expression in the small intestine, indicating local suppression of vitamin D signaling. A high P diet prompted increased mRNA abundances of *CYP27B1* for local vitamin D synthesis, specifically in bone. For FGF23 signaling, analyses revealed ubiquitous expression of *FGFR1-4*, whereas *KL* was expressed in a tissue-specific manner. Dietary P supply did not affect skeletal *FGF23*; however, *FGFR4* and *KL* showed increased expression in bone at high P supply, suggesting regulation to balance mineralization. Specific NRTC responses influence vitamin D metabolism and P homeostasis, which should be considered for a thrifty but healthy P supply.

## 1. Introduction

Maintaining calcium (Ca) and phosphorus (P) homeostasis is crucial for all mammals, including pigs, to enable proper bone metabolism, growth processes, and cellular functions. Due to physiological turn-over, the organism excretes Ca and P, which must be continuously replaced by a diet that meets mineral requirements [1]. However, the availability of minerals in the organism is strictly regulated in mammals, with the kidneys, liver, bones, and intestines specifically interacting in a complex manner to meet the metabolic demand at each stage of development [2].

Key regulators of mineral homeostasis include vitamin D and fibroblast growth factor 23 (FGF23). In particular, the secosteroid hormone calcitriol [1,25(OH)_2_D_3_], the most potent natural metabolite of vitamin D, acts on numerous tissues for both calcemic and non-calcemic effects, although intestinal Ca and P absorption, bone metabolism, and renal mineral reabsorption are the primary subjects of its regulation [2,3]. Calcitriol is formed from precursors by sequential hydroxylation. This involves the conversion of cholecalciferol via CYP2R1 and CYP27A1 in the liver and the subsequent conversion of calcidiol via CYP27B1 in the kidneys (Table 1). Moreover, calcitriol levels are also subject to CYP24A1-mediated degradation [4]. An additional autocrine regulation is known to be present at peripheral sites in non-renal tissues and cells (NRTC) such as the lungs, skin, or parathyroid glands [5]. However, the eventual secretion of non-renal calcitriol into the blood circulation and transport via vitamin D binding protein (DBP, encoded by *GC*) or its autocrine action via the vitamin D receptor (VDR) is still unclear [6]. In conventionally farmed pigs, enteral intake of precursors ensures the subsequent formation of calcitriol [7] although there are also approaches using the UV-B light regimen [8]. Vitamin D metabolism is tightly interlinked with fibroblast growth factor 23 (FGF23) signaling. In mammals, FGF23 is a hormone produced primarily by osteoblasts and osteocytes [9], leading to a decrease in both systemic and non-renal calcitriol production through suppression of *CYP27B1* and induction of *CYP24A1* [10,11]. In kidney, FGF23 reduces P reabsorption and increases urinary P excretion by down-regulating the expression of sodium-dependent phosphate co-transporters in the proximal tubule [12]. The physiological function of FGF23 is mediated by its interaction with protein-encoding FGF receptors (FGFR1–4) and αKlotho (KL), which serves as a transmembrane or soluble co-receptor [13,14]. In addition to its control of mineral homeostasis, FGF23 also exerts KL-independent autocrine and paracrine effects on cytokine secretion, such as in the liver [15].

Knowledge of the regulation of mineral homeostasis in mammals is currently reaching to a new level. The evidence continues to grow for the direct actions of vitamin D and FGF23 and their interactions, with peripheral tissues triggering a variety of non-calcemic functions, including immunity and cell development, as reviewed elsewhere [16,17]. In fact, FGF23 is evolving from a biomarker of disturbed P balance to a pathogenic factor [18]. For pigs, recent dietary intervention studies established the conclusive pattern of endocrine control following variable dietary P supplies [19]. Transcriptome profiles in respective target tissues such as the kidneys and jejunum [20] and parathyroid glands [21] showed endogenous responses in maintaining mineral homeostasis, which consistently highlighted the vitamin D system and associated regulatory pathways. Notably, the assessment of effective mechanisms of mineral homeostasis in pigs is usually based on single measurements that include blood levels of P, Ca, and calcidiol, and therefore do not allow comprehensive conclusions on tissue-level specificities. The expression of all currently annotated sodium-dependent phosphate co-transporters in pigs showed a tissue-specific pattern, with adaptive responses to low dietary P intake compared with high dietary P intake, i.e., increased *SLC34A1* and *SLC34A3* abundances in kidney cortex and reduced *SLC20A2* abundances in the jejunum [22].

**Table 1:** Studied genes involved in vitamin D metabolism and FGF23 signaling.

Gene	Ensembl ID (v. 102)	Description	Function
*CYP2R1*	ENSSSCG00000013389	Cytochrome P450, family 2, R1	Hydroxylation (25-OH) of cholecalciferol (liver) * [23]
*CYP27A1*	ENSSSCG00000016199	Cytochrome P450 family 27, A1	Hydroxylation (25-OH) of cholecalciferol (liver) * [24]
*CYP27B1*	ENSSSCG00000028637	Cytochrome P450 family 27, B1	Hydroxylation (1α-OH) of calcidiol (kidney) * [25]
*CYP24A1*	ENSSSCG00000007486	Cytochrome P450 family 24, A1	Hydroxylation (24-OH) of calcidiol and calcitriol (kidney) * [25]
*VDR*	ENSSSCG00000020864	Vitamin D receptor	Transcription factor [26]
*GC*	ENSSSCG00000027609	Vitamin D binding protein	Binding of calcitriol [27]
*FGF23*	ENSSSCG00000052449	Fibroblast growth factor 23	Regulator of P homeostasis [28]
*FGFR1*	ENSSSCG00000015815	Fibroblast growth factor receptor 1	Receptor of FGF23 and other FGFs [29]
*FGFR2*	ENSSSCG00000010698	Fibroblast growth factor receptor 2	Receptor of FGF23 and other FGFs [29]
*FGFR3*	ENSSSCG00000030827	Fibroblast growth factor receptor 3	Receptor of FGF23 and other FGFs [29]
*FGFR4*	ENSSSCG00000014047	Fibroblast growth factor receptor 4	Receptor of FGF23 and other FGFs [29]
*KL*	ENSSSCG00000009347	Klotho	Co-receptor of FGF23 [30]

* site of hydroxylation; *CYP2R1*, Cytochrome P450 Family 2 Subfamily R Member 1; *CYP27A1*, Cytochrome P450 Family 27 Subfamily A Member 1; *CYP27B1,* Cytochrome P450 Family 27 Subfamily B Member 1; *CYP24A1*, Cytochrome P450 Family 24 Subfamily A Member 1.

Expanding on these findings [22], the present study examines the tissue-specific expression of genes involved in vitamin D metabolism and FGF23 signaling following a conventional standard diet (trial 1) and a P-divergent diet (trial 2). The mRNA expression of a set of genes encoding components of vitamin D metabolism and signaling pathways, as well as FGF23-signaling pathways (Table 1), was investigated in a number of tissues of systemic mineral homeostasis (kidney, bone, various intestinal segments), as well as in additional peripheral tissues (lung, liver, aorta), to gain basic insight into the potential abilities of these tissues to participate in the production, transduction, and elimination of vitamin D metabolites and in the response to FGF23 and its (co)receptors at the very basal level of transcription.

## 2. Results

The study was conducted in pigs, a valuable biomedical model that shares many similarities with humans in its physiology, and at the same time is a target species for improving phosphorus efficiency for more animal and environmentally friendly husbandry [31,32]. Therefore, animals fed a standard conventional diet (experiment 1) or variable P diets (experiment 2) were used to quantify the abundance of transcripts of genes encoding components of vitamin D metabolism and signaling pathways, as well as FGF23-signaling pathways in a variety of tissues, which was complemented by measuring serum calcitriol concentrations.

### 2.1. Tissue-Specific Expression of Genes Linked to Vitamin D Metabolism and FGF23 Signaling under Conventional Standard Dietary P Intake

**Figure 1:**
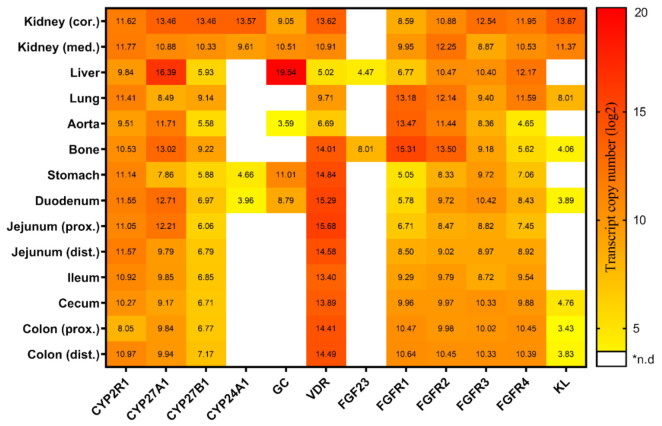
Heatmap illustrating the tissue-specific expression of the key genes linked to vitamin D metabolism and FGF23 signaling in pigs fed on a conventional standard diet (trial 1). Transcript copy numbers of 12 genes were evaluated in 14 tissues by RT-qPCR. The copy numbers were displayed as log2 values and were indicated by a color scale from yellow to red. *n.d, not detectable; cor, cortex; med, medulla; prox, proximal; dist, distal.

The serum calcitriol concentration in pigs fed the conventional diet was 384.5 ± 82.5 pmol/L (mean ± SD) at about 180 days of age. The expression of 12 key genes involved in the synthesis, transport, and metabolism of vitamin D and FGF23 signaling on 14 different tissues is summarized in Figure 1. The hydroxylating enzymes encoding *CYP2R1, CYP27A1,* and *CYP27B1* showed a tissue-wide expression across the investigated panel, whereas the expression of *CYP24A1* was exclusively detectable in the kidneys, stomach, and duodenum. Similarly, *GC* profiles revealed a tissue-specific distribution with its highest transcript abundance in the liver. Moreover, *GC* was found to be expressed in the kidney cortex, kidney medulla, stomach, duodenum and to a lower extent in the aorta. *VDR* was present throughout the tested tissues, but with considerably lower expression in the liver and the aorta. Among the genes related to FGF23 signaling, the expression of *FGF23* and *KL* was shown to be restricted to specific tissues such as kidneys, lung, bone, and the distal intestine, whereas FGF receptors were detectable in all tissues studied. The expression of the *FGF23* transcript was limited to bone and liver. *FGFR1* expression was the highest in bone and showed considerable variation in transcript abundance in the other analyzed tissues. Interestingly, *FGFR2* showed a very similar tissue-specific pattern, with slightly less variation between tissues. In contrast, *FGFR3* profiling revealed an almost constitutive expression in all tissues examined. The highest expression of *FGFR4* was found in the liver; however, considerable amounts were also found in the kidneys, lungs and the intestine. Finally, a high expression of *KL* was noticed in the kidney cortex, kidney medulla, and lung. In general, it was found that almost all genes for vitamin D metabolism and FGF23 signaling were found to be expressed in a considerable and similar extent in the kidney cortex and medulla.

### 2.2. Changes in the Expression of Genes Linked to Vitamin D Metabolism and FGF23 Signaling as a Result of Divergent Dietary P Intake

**Figure 2:**
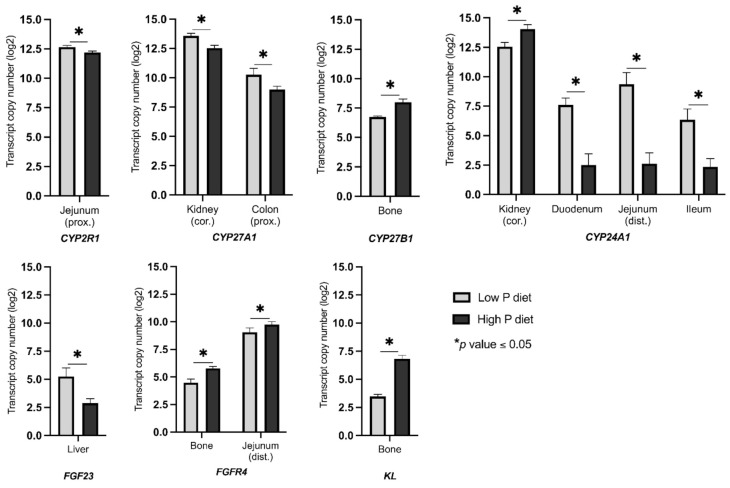
Transcript abundance (copy number) of differentially expressed genes related to vitamin D metabolism and FGF23 signaling in pigs receiving diets with divergent P levels from weaning until 120 days of life (trial 2). Asterisks indicate statistical significance between dietary groups (* *p* ≤ 0.05). cor, cortex; prox, proximal; dist, distal.

The effectiveness of dietary treatment in stimulating regulatory circuits to maintain P homeostasis was demonstrated by analysis of serum calcitriol levels. Following the divergent P diets, calcitriol levels were 751.1 ± 112.4 pmol/L (mean ± SD) on a high-P diet, and 1079.3 ± 134.0 pmol/L on a low P diet, indicating a significant difference (*p* = 0.0047) at 120 days of life. The mRNA expression of a number of the key genes responded in a tissue-specific manner to variable dietary P intake (Table S1, Figure 2). Specifically, the expression of *CYP2R1* significantly differed in proximal jejunum (L > H; FC = 1.38). Another gene encoding one of the 25-hydroxylating enzymes, *CYP27A1*, illustrated a considerable variation in mRNA expression in the kidney cortex (L > H; FC = 2.06) and proximal colon (L > H; FC = 2.39). Furthermore, the vitamin D activating enzyme (1α-hydroxylation) encoding gene *CYP27B1* showed a significant variation in mRNA expression in bone (H > L; FC = 2.39). *CYP24A1*, which encodes for calcitriol 24-hydroxylase and therefore calcitriol elimination, demonstrated the highest response to the P-divergent diet particularly in duodenum (L > H; FC = 34.78), distal jejunum (L > H; FC = 106.52), and ileum (L > H; FC = 16.22). Additionally, a significant change in the mRNA abundance of *CYP24A1* was detected in kidney cortex (H > L; FC = 2.79). *FGF23* exhibited a considerable change in mRNA abundance in liver (L > H; FC = 5.21). Among the *FGF23* receptors, *FGFR4* showed significant differences in mRNA abundances in bone (H > L; FC = 2.45) and distal jejunum (H > L; FC = 1.62). Expression of co-receptor *KL* differed significantly in bone (H > L; FC = 10.16) between the two dietary groups. Among the tissues studied, the expression pattern of bone showed adaptive responses of both vitamin D metabolism and FGF23 signaling after varying dietary P intake.

## 3. Discussion

In monogastric vertebrates, mineral homeostasis is maintained by the tight regulation of intestinal absorption, osseous mobilization, and renal excretion rates involving a number of known and yet to be identified regulators, transporters, and endocrine and paracrine signals. In addition, the complex interplay to maintain mineral homeostasis includes meeting the specific requirements of peripheral NRTC. This involves pathways of mineral metabolism, as well as non-calcemic biological functions of the vitamin D system and FGF23 signaling.

### 3.1. Status Quo and Reactivity of the Vitamin D System to Maintain Mineral Homeostasis

In pigs, expression of the hydroxylases *CYP2R1*, *CYP27A1*, and *CYP27B1* was evident in all tissues examined, which matches previous findings in humans and rodents [5,33,34]. However, in pigs, the mRNA abundance of *CYP2R1* is higher in the kidney cortex and medulla than in the liver, which is in contrast to other animal species [33,34]. Indeed, CYP2R1 has been shown to work in close physical proximity to 1α-hydroxylase, which may explain the presence of *CYP2R1* transcripts in the kidney as the main site of calcitriol production [35]. For *CYP27A1*, the highest mRNA abundance was found in the liver of pigs and other species such as mice [33]. While CYP27A1 shows some activity in 25-hydroxylation, it plays an important role in 27-hydroxylation of cholesterol and bile acid synthesis, i.e., processes that are clearly attributed to the liver [35,36]. Interestingly, CYP27A1 has been further proposed to be involved in 25-hydroxylation of vitamin D_3_ also in kidney [37]. CYP27B1 represents the gatekeeper for the renal activation of systemically acting calcitriol and accordingly it is highly abundant in the renal cortex of pigs. In fact, several modulators including PTH regulate *CYP27B1* expression in kidney, whereas in NRTC, its mRNA abundance may be independent of PTH action [38]. The mRNA abundance of *CYP27B1* in the bone of pigs was substantial, which is in agreement with findings in human and mouse osteocytes and osteoclasts [39,40]. Moreover, *CYP27B1* showed a relatively high mRNA abundance in lung tissue samples. Here, it is assumed that lung epithelial cells and macrophages depend on calcitriol, which triggers immune effects against microbial and viral infections via antimicrobial peptides and cytokines, as well as by initiating immune signaling cascades [41]. Taken together, the tissue-wide distribution of *CYP2R1*, *CYP27A1* and *CYP27B1* in pigs suggests that not only liver and kidney but also peripheral tissues might have the ability to produce calcidiol and calcitriol, which is in agreement with the previous findings in human and mice [42,43]. However, the extent and eventual secretion of calcitriol into the circulation or its autocrine effect on gene expression are still unclear. It has been argued that calcitriol of non-renal origin elicits a variety of effects that are both connected to and independent of mineral homeostasis, including effects on the immune system [41] and control of cell growth and differentiation processes [6]. Calcitriol levels are further modulated via a catabolic enzyme encoded by *CYP24A1*. In pigs, *CYP24A1* showed high expression in the renal cortex and exhibited low mRNA abundances in the stomach and duodenum. Despite the fact that the renal CYP24A1 enzyme helps to balance systemic calcidiol and calcitriol levels, its expression in peripheral tissues is likely to precisely adjust cellular hormone levels in a negative feedback loop [44]. Furthermore, the transport of vitamin D metabolites via the bloodstream is facilitated up to 90% by vitamin D binding proteins (DBP, encoded by *GC*) with different binding affinities (calcidiol > calcitriol > cholecalciferol) [45]. In pigs, *GC* demonstrated the highest mRNA abundance in the liver, which is also the production site of this protein and corresponds to physiologic conditions in humans [46,47]. Consistent with the results, *GC* showed low mRNA abundances in a number of tissues in mice, including the kidneys and intestine [48]. Interestingly, the expression of *GC* in tissues other than the liver has been associated with binding of fatty acids, chemotaxis, binding of endotoxins and impact of T cell response [49]. In pigs, the vitamin D receptor (*VDR*) showed high mRNA abundances in all analyzed tissues. Its ubiquitous expression highlights the fact that vitamin D plays a key role in every single tissue in mammals. Indeed, VDR exerts vitamin-D-dependent and -independent effects in a tissue-specific manner and controls a large number of target genes via vitamin D response elements (VDRE) in the respective promotor region [50]. The high mRNA abundance of *VDR* in the small intestine, bone and kidney demonstrates its involvement in balancing mineral absorption, retention and re-absorption in extracellular fluids [51,52]. Furthermore, novel actions of the calcitriol-VDR complex have been described, e.g., on cell differentiation and proliferation, immune system functions and intracellular signaling cascades [53,54,55].

Indeed, the results demonstrated the effect of divergent dietary P supply on serum calcitriol, with higher levels in L animals compared with H animals. This matches previous findings in growing pigs, but also other species [19,56]. Interestingly, the dietary responses were less pronounced at the major tissue sites known for endocrine synthesis, as the gene expression for *CYP2R1* and *CYP27A1* in the liver as well as for *CYP27B1* in the kidneys did not differ between the experimental groups. In fact, *CYP27B1* showed decreased mRNA abundances in the kidneys of pigs after both low P diets [20] and diets with reduced P and Ca levels [32] compared with control animals. However, the present pig trial showed a marked increase in *CYP24A1* expression in the kidney, suggesting calcitriol elimination in H animals which contributes to the prevention of hypercalcemia and hyperphosphatemia. Therefore, the renal expression pattern of *CYP27B1* and *CYP24A1* represents the result of reciprocal effects, likely mediated by calcitriol, FGF23, and PTH in the kidneys [57]. Thus, calcitriol and FGF23 induce renal *CYP24A1* and suppress renal *CYP27B1*, whereas opposite effects have been reported for PTH [58,59]. It is conceivable that the feedback loops of calcitriol were masked by PTH and FGF23, which in sum favored *CYP24A1* expression in H animals compared with L animals with unchanged renal *CYP27B1* mRNA abundances. In addition, some of the intestinal sections and kidney responded to dietary P supply, as shown by profiles of *CYP2R1* in the proximal jejunum and *CYP27A1* in the proximal colon and renal cortex. Moreover, H animals showed increased *CYP27B1* mRNA abundance in bone. Results suggest a physiological demand for local calcitriol synthesis as a bone-specific response of H animals to compensate for lower endocrine calcitriol concentrations in serum compared with L animals. Reduced osteoclastogenesis has been shown previously in mutant mice lacking *CYP27B1* expression in chondrocytes [60]. Conversely, a transgene strain overexpressing *CYP27B1* in chondrocytes exhibited lowered bone volume and the trabecular number [60]. This supports previous results on physiology in pigs that showed that microstructural bone characteristics were found to plateau with increasing dietary P supply [19]. In the small intestine, results revealed regulatory mechanisms to reduce calcitriol levels in L animals compared to H animals. The inducible character of intestinal *CYP24A1* after calcidiol injection [61] suggests that high *CYP24A1* mRNA abundances in duodenum, jejunum, and ileum might help to precisely control cellular calcitriol levels. Specifically, systemic calcitriol levels can directly affect intestinal mineral absorption processes, e.g., by regulating *SLC34A3* and *TRPV6* expression [22,62], which are counterbalanced at the local level by calcitriol elimination to prevent hypercalcemia in L animals. Indeed, activity of CYP24A1 has been shown to be targeted via negative feedback loops of calcidiol and calcitriol [63]. In the context of respective endocrine serum levels, control of anabolic and catabolic enzymes related to calcitriol and vitamin D metabolism is important for maintaining serum P and Ca levels and tissue integrity. Regarding the transport of vitamin D metabolites, the *GC* mRNA abundances were unaltered by variable dietary P supply in porcine NRTC. The results match current reports in humans in which high dietary vitamin D levels had no effect on GC protein abundances [64]. Although GC expression has been reported to be dependent on TGFβ, IL-6, and glucocorticoids [65], the associated physiological states of sexual maturation and inflammation are not present in pigs at 120 days of age.

### 3.2. Systemic and Autocrine Regulations of FGF23 Signaling

In pigs, FGF23 expression is highly tissue-specific and primarily restricted to bone and also to liver. It has been shown that the liver has the capacity to express FGF23 in mice [66], especially in an inflammatory state [67,68]. Numerous studies have shown that the kidney is the main target organ of FGF23, with effects on regulation of P reabsorption triggered by activation of the mitogen-activated protein kinase (MAPK) cascade [69]. Moreover, FGF23 has been associated with an increasing number of side effects in other tissues [70]. Similarly to kidney, binding between FGF23 and the FGFR-KL receptor complex is also required in NTRC to mediate downstream effects, although KL-independent cascades have been reported [71]. The ubiquitously expressed *FGFR1-4* receptors in pigs indicate a tissue-wide capacity for FGF23 signal transduction. The highest mRNA abundances for *FGFR1* and *FGFR2* were found in bone, suggesting its involvement in osteoblast proliferation and bone formation [72,73]. Moreover, functions of these two FGF receptors are conceivable in the aorta as well as in the lung, where the pig analysis also revealed high abundances of *FGFR1* mRNA [74,75]. Interestingly, *FGFR1* transcripts were also detected in the intestine, where ascending mRNA abundances along the gastrointestinal sections were observed. An effect of FGF23 on the intestine is attributed to indirect effects via lowering of calcitriol levels and, consequently, P absorption [76]. Although *FGFR3* and *FGFR4* show high mRNA abundances in the renal cortex, they are thought to make little or no contribution to renal FGF23 effects [12,77]. However, other ligands such as FGF19 and FGF21 have been shown to interact with FGFR1-4 in a tissue-specific manner to regulate, e.g., fat metabolism and bile acid synthesis [78]. The important co-receptor *KL* appeared to be expressed in a relatively tissue-specific manner with the highest mRNA abundances in both the kidney cortex and the medulla [79,80]. This supports the assumption that the kidney acts as the primary target site of FGF23 in pigs, the same as in other mammals, to maintain P homeostasis [81]. Furthermore, the subtle *KL* expression in intestinal sections such as the duodenum and colon points to functions of FGFs in an autocrine manner [82]. In addition to the membrane-bound form of KL, its soluble form released into the circulation is known to trigger both FGF23-mediated and FGF23-independent responses in NRTC [14,83]. Besides mediating mineral metabolism, the occurrence of soluble KL is currently associated with cell-protective functions, such as the inhibition of apoptosis, senescence, and oxidative stress [72,84,85], i.e., molecular themes that are also attributed to membrane-bound KL in the lung and alveolar cells, which showed relatively high *KL* mRNA abundances in pigs [86,87].

Transcriptional responses due to variable dietary P supply showed unaltered mRNA abundances of the *FGFR* and *KL* in porcine kidneys. Furthermore, *FGF23* expression in the bones of pigs was unaltered between dietary groups. However, it has been reported elsewhere that a low dietary P supply decreases FGF23 protein secretion and therefore a lowered renal P excretion [88]. Interestingly, L animals showed higher *FGF23* mRNA abundances in liver tissue than H animals. It is conceivable that results represent adaptive responses as the liver has been shown previously to express *FGF23* following Jak1/Stat3-induced local inflammation [68]. In mice, hepatic *FGF23* expression was accompanied by increased levels of serum calcitriol, decreased PTH, and unaltered levels of FGF23 derived from bone [68], revealing another potential mechanism for the tissue-wide response to variable dietary P supply. Importantly, both the intact protein (iFGF23) and the c-terminal cleavage product (cFGF23), which lacks phosphaturic activity, can be secreted [84]. Due to the latter, FGF23 is currently being discussed as a biomarker for pathophysiological implications in nephrology and cardiology, although its reliable translation into clinical utility is still pending [85]. In this context, FGF23 has been demonstrated to induce the production of inflammatory cytokines such as TNF-α and IL-6 in the liver, highlighting FGFR4 as a therapeutic target [15]. Regarding FGF23 signal transduction, *FGFR4* and *KL* in bone showed significant diet-dependent transcriptional effects with increased mRNA abundance in H animals compared with L animals. As FGFR4 and KL are key regulators of osteogenesis affecting the differentiation and function of osteoblasts [89,90], transcriptional responses might be attributed to balance bone mineralization in H animals as a part of potential autocrine regulatory circuits [91].

## 4. Materials and Methods

### 4.1. Animals and Diets

Animal trials used for this study were approved by the Scientific Committee of the Research Institute for Farm Animal Biology (FBN). The experimental setup was licensed and endorsed by the ethics committee of the federal state of Mecklenburg-Western Pomerania, Germany (Landesamt für Landwirtschaft, Lebensmittelsicherheit und Fischerei). It was registered under the license LALLF M-V/TSD/7221.3-1-053-15 (16 December 2015). The sample set used for this study has been introduced previously [22]. In trial 1, five German Landrace fattening pigs (two females, three castrates; purebred animals of the species Sus scrofa domesticus) were fed ad libitum a complete standard diet according to the current recommendation [1]. The animals reached an average body weight of 118.4 ± 1.7 kg at slaughter (6 months of age). In trial 2, a total of ten crossbred pigs (German Landrace × Large White × Pietrain; crossbred animals representing typical slaughter pigs) of three litters were supplemented with P-divergent diets from weaning (28th day of life) until they were slaughtered (4 months of age) at an average body weight of 90.7 ± 4.6 kg (L) and 99.8 ± 12.3 kg (H) (mean ± SD). Among them, five piglets (three males, two females) were fed low (L) phosphorus (P) diets and five piglets (three males, two females) were fed high (H) P diets [22]. At the juvenile ages used, no differences were found between male, female and castrated animals with regard to P homeostasis. In the grower period (28th–70th day of life), the fed dietary P and Ca levels were 5.2 and 9.8 g/kg (L), and 7.8 and 9.1 g/kg (H). The finisher (71st–120th day of life) diet had 4.1 and 6.5 g/kg (L), and 7.0 and 6.7 g/kg (H) of P and Ca (Table S2). Neither phytase nor other phosphatases were included in the diet. The pigs were given ad libitum access to pelleted feed and water.

### 4.2. Tissue and Serum Sampling

The tissue sampling has been described previously [22]. In the slaughterhouse at FBN, the pigs were anesthetized using electrical stunning and sacrificed by exsanguination. A total number of 14 tissues (trial 1) and 11 tissues (trial 2) were sampled according to Table 2. Prior scraping, the mucosa was washed with ice-cold saline solution (0.9% NaCl) to remove any residual digesta.

The samples were prepared by cutting them into pieces and freezing in liquid nitrogen immediately. Additionally, trunk blood was collected, clotted for about 30 min, and centrifuged (3500× *g*; 10 min) to prepare serum. All samples were kept frozen at −80 °C until downstream analysis.

### 4.3. RNA Isolation and cDNA Synthesis

The total RNA was extracted from all tissue samples using TRI reagent according to the user’s guidelines (Sigma-Aldrich, Taufkirchen, Germany), and treated with DNase1 to ensure the removal of any residuals of genomic DNA. The RNA samples were purified using the column-based NucleoSpin RNA II-kit (Macherey-Nagel, Düren, Germany). The final concentration of the purified RNA was measured using the NanoDrop 2000 Spectrophotometer. The absence of genomic DNA in total RNA samples was checked by PCR amplification with beta-actin (*ACTB*)-specific primers (Table 3). First-strand cDNA was synthesized from total RNA using random primers (Promega, Fitchburg, WI, USA) and oligo d(T) primers in the presence of SuperScript III reverse transcriptase (Invitrogen, Karlsruhe, Germany). The absence of genomic DNA contamination was checked following PCR amplification of porcine *ACTB* with intron-spanning primers.

### 4.4. Quantitative Real-Time PCR

The primers of all target genes (Table 3) were designed using the sequence information from the Ensembl database (https://www.ensembl.org; accessed on 18 January 2021) and the NCBI primer blast online tool (https://www.ncbi.nlm.nih.gov/tools/primer-blast; accessed on 18 January 2021). The performance of primers and an initial evaluation of expected amplification conditions were assessed by PCR using SupraTherm Taq Polymerase (GeneCraft, Lüdinghausen, Germany) with standard cycling conditions (initial denaturation at 95 °C for 3 min, followed by 40 cycles consisting of denaturation at 95 °C for 15 s, annealing at corresponding annealing temperature for 30 s, and extension at 72 °C for 60 s with a final extension at 72 °C for 5 min). For the preparation of the initial standards for the standard curve, the respective amplificates were visualized after electrophoresis on an agarose gel, purified with magnetic beads (Beckmann Coulter, Krefeld, Germany) and measured with the NanoDrop 2000. The expression levels of target genes and *RPL32* (housekeeping gene) were quantified using quantitative real-time qPCR. The transcript copy numbers were measured in duplicate using the LightCycler 480 SYBR Green I master mix (Roche, Mannheim, Germany) according to the user’s guidelines. In detail, the reaction mix contained the following: 6 µL of SYBR Green Master I mix, 0.6 µL of each primer (10 µM), 2.8 µL of nuclease-free water, and 2 µL of cDNA. Using the LightCycler480 system (Roche), the PCR amplification program was set as follows: 95 °C for 5 min, followed by 45 cycles of 95 °C for 10 s, 60 °C for 15 s, and 72 °C for 25 s. Finally, a melting curve analysis was performed to evaluate the amplified products. The transcript copy numbers of each sample were revealed based on the standard curve method, which utilizes the cycle threshold values of serial dilutions (10^7^–10^0^ copies) to the corresponding standard.

### 4.5. Serum Measurement of Calcitriol

The serum concentration of calcitriol [1,25(OH)_2_D_3_] was measured in duplicate for all samples of trial 1 and trial 2 using a commercially available enzyme-linked immunosorbent assay (ELISA) kit (AC-62F1, Immunodiagnostic Systems GmbH, Frankfurt am Main, Germany) according to the manufacturer’s protocol.

### 4.6. Data Analyses

The data analyses of this experiment were performed using the open-sourced R software (v4.1.1; R foundation for statistical computing, Vienna, Austria). For gene expression analysis, the transcript copy numbers were factorially normalized based upon the expression of the housekeeping gene *RPL32* and transformed log2. To be considered for further analysis, the mean log2 copy number of duplicates had to be above 2.5 for at least 50% of the samples in each tissue. The tissue-specific transcript copy numbers (mean value) were visualized by a heatmap using GraphPad Prism v9.2.0 (GraphPad software, San Diego, CA, USA). For trial 2, a linear model (R package stats v4.1.1; R foundation for statistical computing, Vienna, Austria) was used to compare the gene expression and serum calcitriol concentrations between dietary groups. Sex was used as a fixed effect. Differences at *p* ≤ 0.05 were considered statistically significant. Fold changes (FC) have been calculated based on mean expression values between the two dietary groups.

## 5. Conclusions

The ubiquitous distribution of *CYP2R1*, *CYP27A1*, and *CYP27B1* in pigs suggests a tissue-wide capacity for systemic and local calcidiol and calcitriol synthesis. In contrast, *CYP24A1* expression and thus calcitriol clearance appeared to be site-specific and occur at normal P supply only in the kidneys. Nevertheless, the significant response of intestinal *CYP24A1* due to variable P diets demonstrated the crucial role of autocrine mechanisms to balance local calcitriol actions. The tissue-wide expression of *VDR* underlines the multifactorial impact of the vitamin D system with an emphasis on intestine. Regarding the involvement of the FGF-system in mineral balance, the ubiquitous distribution of *FGFRs* transcripts implies tissue-wide capacity for signal transduction mediated by FGF23 of bone origin. The significant increase in expression of skeletal *FGFR4* and *KL* following high dietary P supply point to autocrine circuits to regulate bone mineralization. Tissue-wide analyses of the expression of genes encoding enzymes and (co-)receptors of vitamin D and FGF23 metabolism reflect the complex endogenous mechanisms at the level of transcripts and reveal tissue-specific and diet-related expression patterns. These findings warrant further studies to monitor the effects of diets on health and tissue integrity under specific conditions and in selected tissues at the level of protein expression and enzyme activation.

## Figures and Tables

**Table 2:** Tissue samples collected for the pig trials, as previously stated [22].

Tissue Labeling	Description	Trial
Kidney cortex	Cortex of left kidney	1, 2
Kidney medulla	Medulla of left kidney	1, 2
Liver	*Lobulus spigelii*	1, 2
Lung	Lower tip of the left lung lobe	1
Aorta	Aorta, descending thoracic aorta	1
Bone	Calvarial bone along the sagittal suture	1, 2
Stomach	Fundus mucosa	1
Duodenum	Mucosa 30–40 cm distal of pylorus	1, 2
Jejunum (prox.)	Mucosa 2 m distal of pylorus	1, 2
Jejunum (dist.)	Mucosa 2 m proximal of the ileocecal junction	1, 2
Ileum	Mucosa 20 cm proximal of the ileocecal junction	1, 2
Caecum	Mucosa	1, 2
Colon (prox.)	Mucosa 50–60 cm distal of cecolic junction	1, 2
Colon (dist.)	Mucosa 50–60 cm proximal of rectum	1, 2

prox, proximal; dist, distal.

**Table 3:** Primer sequences, annealing temperatures and resulted fragment sizes.

Sl. No.	Genes	Forward Primer (5′-3′)	Reverse Primer (5′-3′)	AT * (°C)	FS ** (bp)
1	*CYP2R1*	TTGCTTCAGCGATTTCACTTG	TGTGCATTTTCAGCGTCTTTC	60	123
2	*CYP27A1*	CAAGTACCCAGTACGGAACGAC	AGCATCCGCTGGTTCAGAG	60	132
3	*CYP27B1*	CCATCAGCCACTGTTCTATCC	TCCCTTGAAGTGGCATAGTGAC	60	179
4	*CYP24A1*	GGAATTGTATGCGTCTGTGAC	CATCTGATTCTCAGGCAGTACAC	60	154
5	*GC*	AAGTTGCCCACAAACAAAGATG	TCAGGGTTGGCTCAAGTATTTTAC	60	130
6	*VDR*	CTTCTGTGACCCTGGACCTG	GCACTTGACTTCAGCAGCAC	60	157
7	*FGF23*	CAGGCTTCGTGGTCATAACAG	CTGACGAGGAAGCGGTAGTG	60	172
8	*FGFR1*	GACTCCTAACCCCACCTTGC	GTGTAGTTGCCCTTGTCGGA	60	141
9	*FGFR2*	CCTCACAGAGACCCACCTTC	GTTCGAGAGGCTGACTGAGG	60	212
10	*FGFR3*	TCATAGGCGTGGCTGAGAAG	CACCACCAGGATGAAGAGGAG	60	187
11	*FGFR4*	AGAGTACCTTGACCTCCGCT	CTCATGGCTGAAGACCGAGT	60	213
12	*KL*	ACTGGCTGAGGTCCAAGTACG	GGAGCTGTGCGATCATTAAATG	60	199
13	RPL32 ***	AGCCCAAGATCGTCAAAAAG	TGTTGCTCCCATAACCAATG	60	165
14	*ACTB*	GAGAAGCTCTGCTACGTCGC	CCTGATGTCCACGTCGCACT	60	231

* Annealing temperature, ** Fragment size at cDNA level, *** Housekeeping gene.

## Data Availability

The data presented in this study are available in the main article and the Supplementary Materials.

## Supplementary Materials

The following supporting information can be downloaded at: www.mdpi.com/article/10.3390/metabo12080729/s1, Table S1: Descriptive statistics and tissue-specific transcript copy numbers of the analyzed candidate genes in response to a variable dietary intake of phosphorus; Table S2: Analyzed nutrient composition of the experimental diets fed to offspring during starter and finisher periods.
