# Supplementary material for: Tissue-Wide Expression of Genes Related to Vitamin D Metabolism and FGF23 Signaling following Variable Phosphorus Intake in Pigs
Source: Metabolites. 2022 Aug 6;12(8):729. doi: 10.3390/metabo12080729 (PMC9413461; doi:10.3390/metabo12080729)
Supplement: Supplementary file 1 [file metabolites-12-00729-s001.zip › metabolites-1842131-supplementary.pdf]

Table S1. Descriptive statistics and tissue-specific transcript copy numbers of the analyzed candidate genes in response to a variable dietary intake of phosphorus.

Genes	Tissue	p-value	FC*	L vs. H**	Transcript copy number (log2)		
					Mean	Min	Max
CYP2R1	Kidney cortex	0.5835	1.10	H>L	11.2	10.6	11.9
	Kidney medulla	0.6192	1.18	L>H	10.8	9.2	11.5
	Liver	0.7030	1.15	L>H	10.2	9.3	11.6
	Bone	0.1289	1.32	H>L	8.7	8.1	9.2
	Duodenum	0.5772	1.09	L>H	12.4	11.9	13.1
	**Jejunum (Prox.)**	**0.0435**	**1.38**	**L>H**	**12.4**	**12.0**	**13.1**
	Jejunum (Dist.)	0.3252	1.13	H>L	12.0	11.7	12.5
	Ileum	0.3652	1.27	L>H	12.1	11.4	13.1
	Cecum	0.3178	1.32	H>L	11.6	10.6	12.3
	Colon (Prox.)	0.6857	1.25	H>L	9.4	8.0	11.3
	Colon (Dist.)	0.2623	1.34	H>L	12.0	11.0	12.6
CYP27A1	**Kidney cortex**	**0.0000**	**2.06**	**L>H**	**13.1**	**12.1**	**14.2**
	Kidney medulla	0.5962	1.27	H>L	10.7	9.0	12.3
	Liver	0.9242	1.01	H>L	16.8	16.2	17.1
	Bone	0.2088	1.46	H>L	11.2	10.7	12.7
	Duodenum	0.6819	1.16	L>H	11.9	10.7	13.0
	Jejunum (Prox.)	0.4500	1.62	L>H	11.7	9.6	13.3
	Jejunum (Dist.)	0.9381	1.03	L>H	10.4	9.3	11.3
	Ileum	0.4076	1.47	L>H	9.8	8.3	11.8
	Cecum	0.4243	1.32	H>L	9.6	8.3	10.7
	**Colon (Prox.)**	**0.0333**	**2.39**	**L>H**	**9.6**	**8.2**	**11.6**
	Colon (Dist.)	0.6896	1.10	L>H	10.5	9.8	11.2
CYP27B1	Kidney cortex	1.0000	1.00	H>L	14.8	14.1	15.5
	Kidney medulla	0.9314	1.07	H>L	10.2	8.2	13.6
	Liver	0.5868	1.23	L>H	6.0	5.0	7.7
	**Bone**	**0.0032**	**2.39**	**H>L**	**7.3**	**6.5**	**8.6**
	Duodenum	0.6865	1.18	H>L	7.0	5.4	7.9
	Jejunum (Prox.)	0.0917	1.82	L>H	6.7	4.9	7.7
	Jejunum (Dist.)	0.7975	1.09	L>H	6.7	5.7	7.7
	Ileum	0.1259	1.20	L>H	7.0	6.6	7.5
	Cecum	0.5330	1.20	H>L	8.0	7.0	8.8
	Colon (Prox.)	0.1812	1.47	L>H	6.7	5.4	7.4
	Colon (Dist.)	0.1020	1.27	H>L	8.0	7.5	8.5
CYP24A1	**Kidney cortex**	**0.0321**	**2.79**	**H>L**	**13.3**	**11.8**	**15.3**
	Kidney medulla	0.7551	1.21	H>L	8.9	6.8	11.4
	Bone	0.3964	2.43	L>H	7.0	2.6	9.3
	**Duodenum**	**0.0028**	**34.78**	**L>H**	**5.1**	**0.5**	**8.6**
	Jejunum (Prox.)	0.2339	11.96	L>H	7.0	1.1	15.1
	**Jejunum (Dist.)**	**0.0030**	**106.52**	**L>H**	**6.4**	**0.6**	**11.2**
	**Ileum**	**0.0140**	**16.22**	**L>H**	**4.4**	**0.8**	**8.4**

	Cecum	0.5859	1.68	L>H	3.3	1.3	5.7
VDR	Kidney cortex	0.0720	1.72	H>L	13.0	12.2	13.8
	Kidney medulla	0.8721	1.09	L>H	10.5	9.0	12.0
	Liver	0.5363	1.15	H>L	4.3	3.8	5.0
	Bone	0.3310	1.14	H>L	12.5	11.9	12.9
	Duodenum	0.1034	1.89	L>H	15.4	13.9	16.9
	Jejunum (Prox.)	0.9756	1.01	L>H	15.8	14.7	17.1
	Jejunum (Dist.)	0.1204	2.03	L>H	14.8	13.9	16.7
	Ileum	0.0591	3.58	L>H	13.8	11.9	17.2
	Cecum	0.9472	1.01	H>L	13.6	12.8	14.3
	Colon (Prox.)	0.1275	1.89	L>H	13.7	12.0	15.1
	Colon (Dist.)	0.0933	1.36	H>L	14.3	13.8	15.2
FGF23	**Liver**	**0.0116**	**5.21**	**L>H**	**4.1**	**2.0**	**7.0**
	Bone	0.1030	2.55	H>L	4.4	2.0	5.5
FGFR1	Kidney cortex	0.1203	1.34	L>H	11.7	10.9	12.4
	Kidney medulla	0.9424	1.03	H>L	13.5	12.1	14.9
	Liver	0.5079	1.16	H>L	10.1	9.5	11.1
	Bone	0.1314	1.51	H>L	14.3	13.7	15.6
	Duodenum	0.6125	1.30	H>L	10.3	8.3	11.7
	Jejunum (Prox.)	0.9424	1.04	H>L	10.2	8.6	11.6
	Jejunum (Dist.)	0.6406	1.20	H>L	10.9	9.4	11.9
	Ileum	0.4649	1.38	H>L	10.2	8.4	11.5
	Cecum	0.8850	1.04	L>H	11.6	10.5	12.6
	Colon (Prox.)	0.1297	1.82	L>H	11.1	9.8	12.4
	Colon (Dist.)	0.2248	1.32	H>L	11.7	11.0	12.4
FGFR2	Kidney cortex	0.3339	1.16	H>L	10.6	10.2	11.2
	Kidney medulla	0.7935	1.06	L>H	12.4	11.8	13.1
	Liver	0.5817	1.16	H>L	10.8	9.8	11.6
	Bone	0.2599	1.24	H>L	12.7	12.1	13.1
	Duodenum	0.1859	1.39	H>L	10.5	9.7	11.1
	Jejunum (Prox.)	0.0547	1.62	L>H	10.6	9.8	11.5
	Jejunum (Dist.)	0.1819	1.20	H>L	11.2	10.3	11.8
	Ileum	0.7358	1.09	L>H	10.4	9.5	11.4
	Cecum	0.6603	1.13	H>L	10.7	9.8	11.6
	Colon (Prox.)	0.0928	1.38	L>H	9.5	8.9	10.8
	Colon (Dist.)	0.2023	1.18	H>L	11.5	10.9	11.9
FGFR3	Kidney cortex	0.1841	1.77	L>H	10.8	8.8	11.9
	Kidney medulla	0.9658	1.01	H>L	9.2	8.3	10.2
	Liver	0.7049	1.12	L>H	10.8	9.5	11.4
	Bone	0.1977	1.19	H>L	9.3	8.8	9.8
	Duodenum	0.1404	1.36	L>H	9.9	9.2	10.6
	Jejunum (Prox.)	0.2231	1.87	L>H	10.1	8.3	11.5
	Jejunum (Dist.)	0.5838	1.15	H>L	10.9	10.3	11.5
	Ileum	0.3814	2.08	L>H	8.7	6.8	11.3
	Cecum	0.7516	1.09	H>L	9.9	8.9	10.7
	Colon (Prox.)	0.1268	1.97	L>H	9.0	7.0	10.7
	Colon (Dist.)	0.2735	1.15	H>L	10.5	10.2	11.0

FGFR4	Kidney cortex	0.6978	1.06	L>H	12.4	12.0	12.9
	Kidney medulla	0.6689	1.15	H>L	11.3	10.4	12.3
	Liver	0.8050	1.04	H>L	12.1	11.6	12.8
	**Bone**	**0.0226**	**2.45**	**H>L**	**5.1**	**3.6**	**6.2**
	Duodenum	0.4862	1.32	H>L	8.4	6.8	9.5
	Jejunum (Prox.)	0.1968	1.72	L>H	8.8	7.4	9.8
	**Jejunum (Dist.)**	**0.0119**	**1.62**	**H>L**	**9.4**	**8.2**	**10.4**
	Ileum	0.4778	1.21	H>L	9.5	8.4	10.1
	Cecum	0.6549	1.15	H>L	9.6	8.5	10.6
	Colon (Prox.)	0.0795	1.67	L>H	9.3	8.0	10.8
	Colon (Dist.)	0.2199	1.20	H>L	9.5	9.1	10.2
GC	Kidney cortex	0.3138	1.56	L>H	11.6	10.1	13.5
	Kidney medulla	0.2027	2.14	L>H	11.4	9.5	13.2
	Liver	0.8525	1.04	L>H	19.8	19.2	20.5
	Bone	0.4665	1.87	L>H	4.6	2.2	7.1
	Colon (Dist.)	0.8471	1.06	H>L	2.7	1.8	3.7
KL	Kidney cortex	0.2612	1.56	L>H	13.9	12.7	15.4
	Kidney medulla	0.3094	1.92	L>H	12.1	10.0	14.4
	Liver	0.1186	1.82	H>L	3.0	1.3	3.9
	**Bone**	**0.0081**	**10.16**	**H>L**	**5.0**	**2.2**	**8.5**
	Duodenum	0.1250	1.58	H>L	6.0	5.0	7.2
	Jejunum (Prox.)	0.0764	2.08	L>H	5.0	3.7	6.3
	Jejunum (Dist.)	0.0576	1.45	H>L	5.2	4.4	6.3
	Ileum	0.6965	1.10	L>H	4.9	3.6	5.5
	Cecum	0.6730	1.15	L>H	6.8	5.9	7.9
	Colon (Prox.)	0.3596	2.39	L>H	3.3	0.4	5.2
	Colon (Dist.)	0.7380	1.09	L>H	5.7	5.0	6.7

*Fold change; **Low (L) phosphorus vs. high (H) phosphorus diet;

Table S2. Analyzed nutrient composition of the experimental diets fed to offspring during grower and finisher periods.

Diets		Grower diets (day 28-70)		Finisher diets (day 71-120)	
**Item**	**Unit**	**L**	**H**	**L**	**H**
DM	g/kg	901	898	880	883
Crude ash	g/kg	62.2	66.3	38.9	44.6
Crude Protein	g/kg	192	188	178	175
Crude fat	g/kg	56.6	56.2	28.8	31.2
Crude fiber	g/kg	37.4	36.1	34.9	38.7
Starch	g/kg	364	357	434	432
ME (pig)	MJ/kg	13.9	13.8	13.5	13.4
Calcium	g/kg	9.80	9.08	6.50	6.70
Phosphorus	g/kg	5.20	7.80	4.12	6.96
Ca:P ratio		1.88	1.17	1.58	0.96
